# Supplementary figures and images for: Golgi associated RAB2 interactor protein family contributes to murine male fertility to various extents by assuring correct morphogenesis of sperm heads
Source: PLoS Genet. 2024 Jun 27;20(6):e1011337. doi: 10.1371/journal.pgen.1011337 (PMC11236154; doi:10.1371/journal.pgen.1011337)

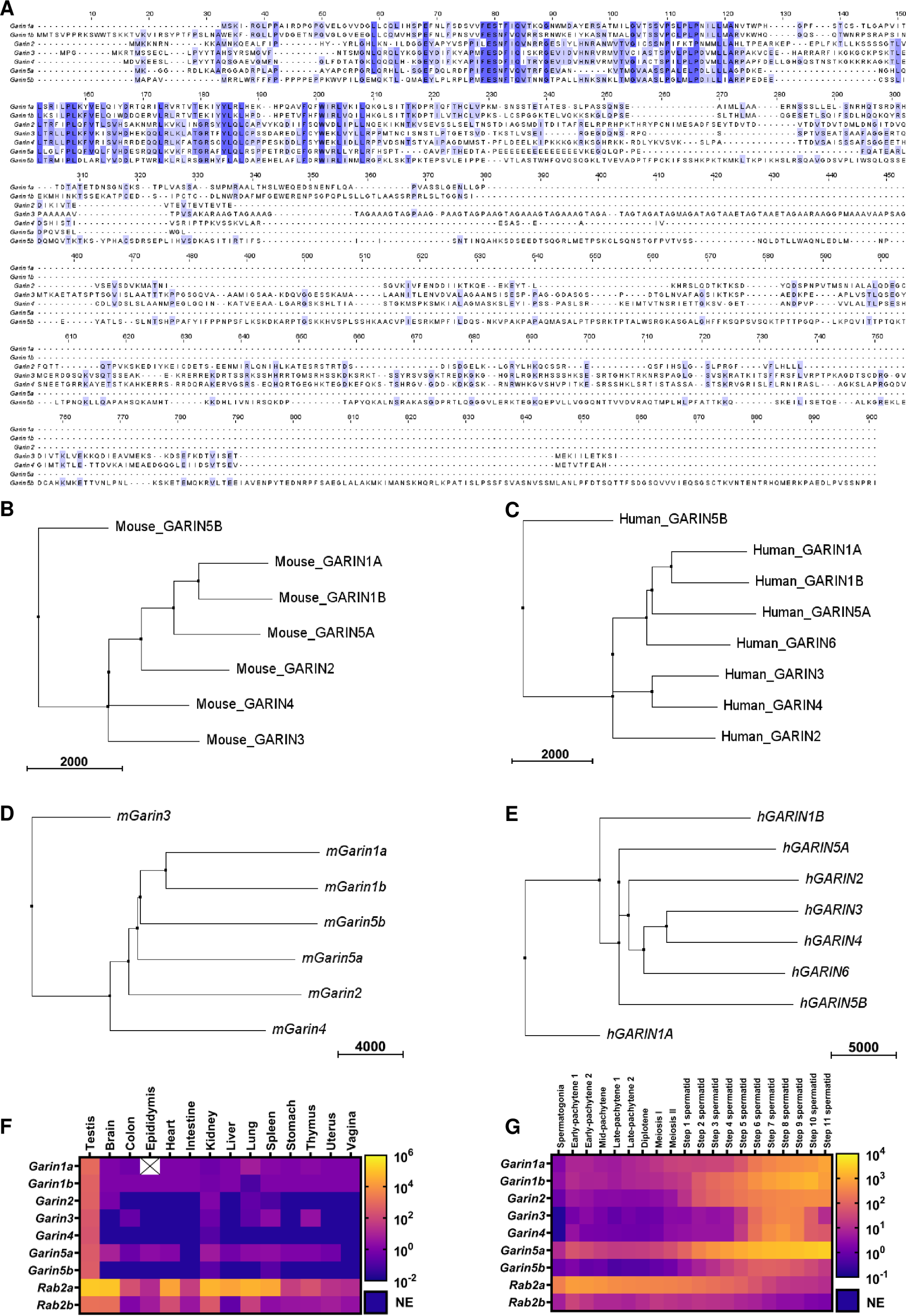

Supplement: S1 Fig — (A) Sequence comparison of GARINs in mice. (B–C) Phylogenetic analyses of amino acid sequences on mouse GARINs (B) or human GARINs (C). (D–E) Phylogenetic analyses of coding sequences on mouse Garins (D) or human GARINs (E). (F) Expression of GARINs and RAB2A/B in mouse tissues. Data was obtained from the Expression Atlas. NE, no expression. (G) Data on the mRNA expression of GARINs and RAB2A/B at different stages of spermatogenesis. (TIF) [file pgen.1011337.s001.tif]

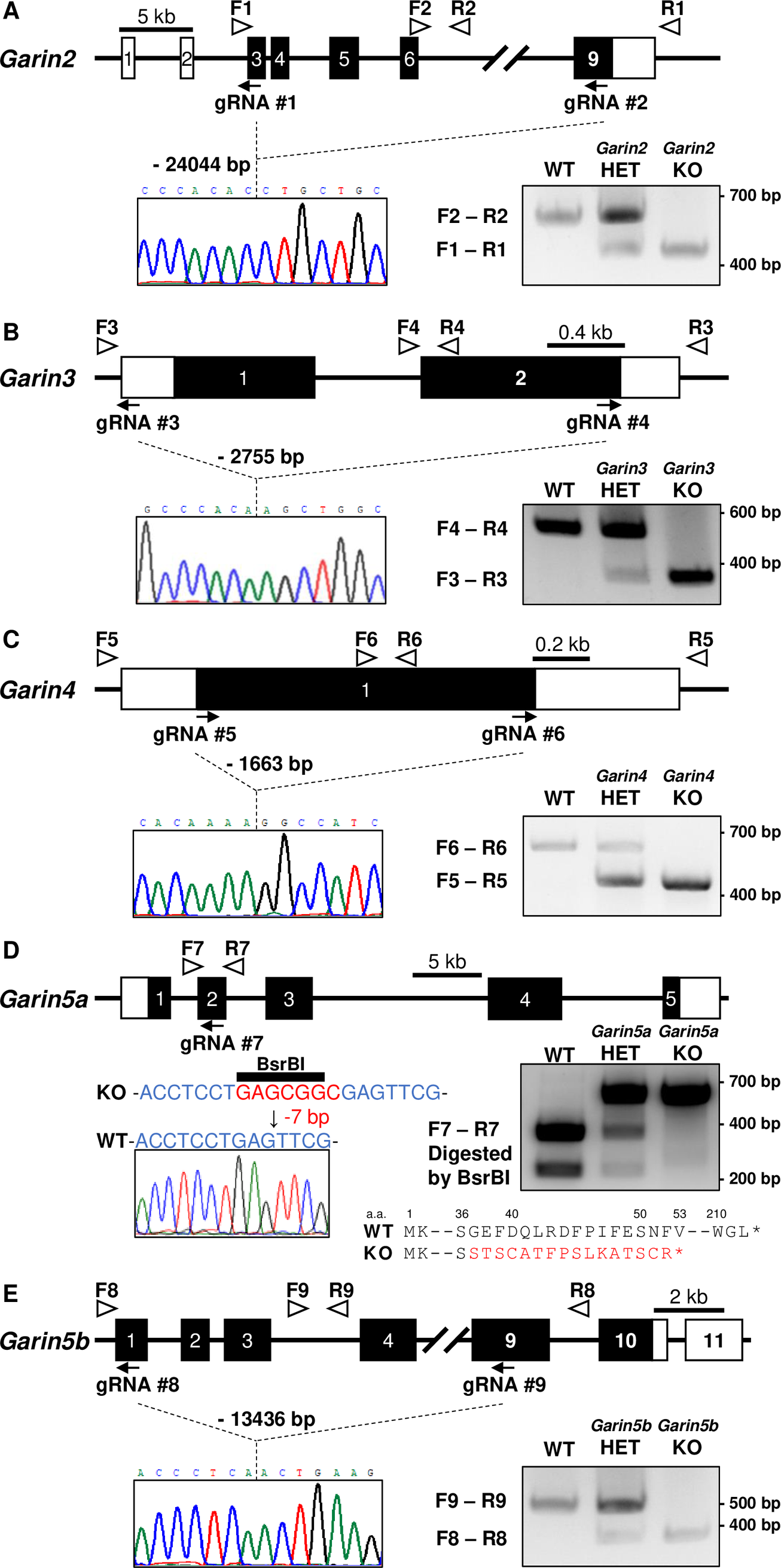

Supplement: S2 Fig — (A–E) KO mouse generation strategy, deletion size, deletion site, and genomic PCR of Garin2 (A), Garin3 (B), Garin4 (C), Garin5a (D), and Garin5b (E). (TIF) [file pgen.1011337.s002.tif]

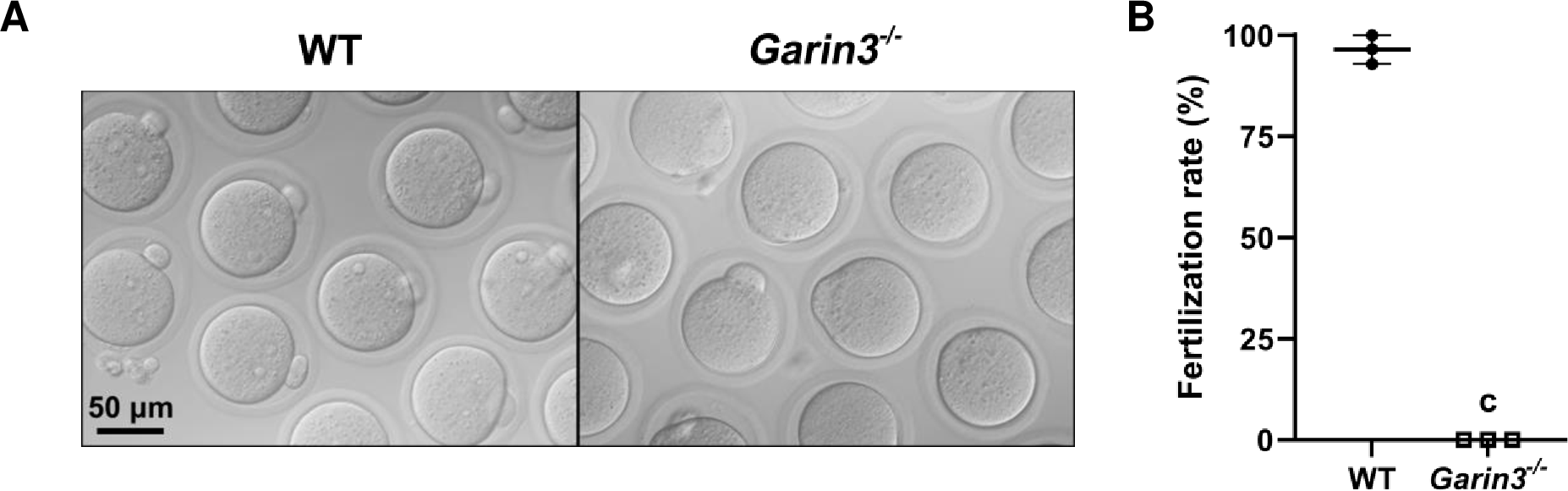

Supplement: S3 Fig — (A) Representative image of eggs collected from WT females that were mated with WT or Garin3-/- males. Eggs were collected 8 hours after mating. (B) 2PN formation rates of eggs collected from WT females which were mated with WT or Garin3-/- males (Student’s t-test, c, P < 0.001). (TIF) [file pgen.1011337.s003.tif]

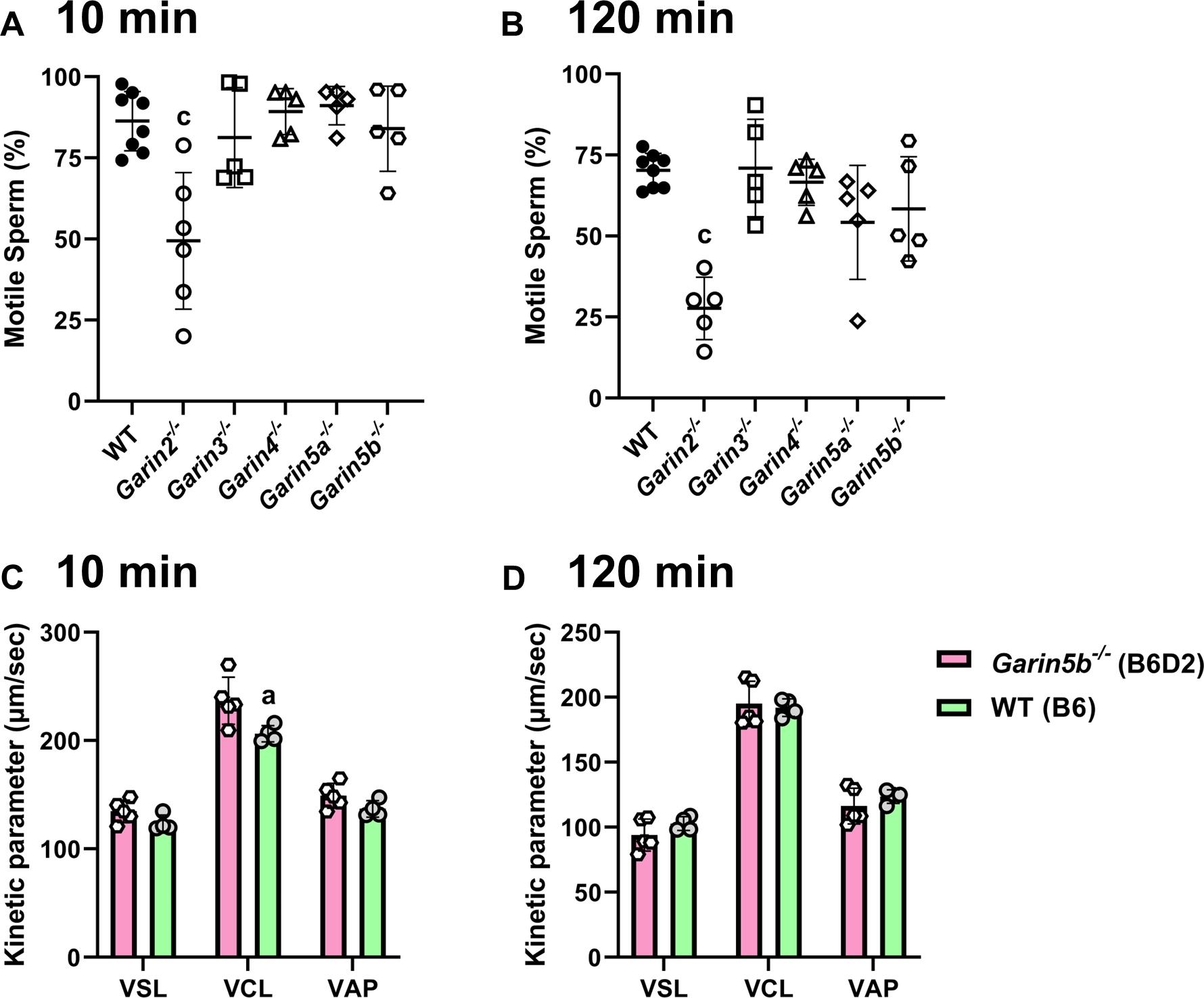

Supplement: S4 Fig — (A–B) Motile sperm rates of WT and Garin2-5b (Garin2, 3, 4, 5a, and 5b) KO mice after incubating for 10 minutes (A) and 120 minutes (B). Garin2-5b-/- groups were compared with the WT group (One-way ANOVA, c, P < 0.001). (C—D) Average of straight line velocity (VSL), curvilinear velocity (VCL), and average path velocity (VAP) of WT spermatozoa [from C57BL/6 (B6) mice] and Garin5b KO spermatozoa [from C57BL/6 × DBA/2 (B6D2) hybrid mice] after incubating for 10 minutes (C) and 120 minutes (D) (Student’s t-test, a, P < 0.05). (TIF) [file pgen.1011337.s004.tif]

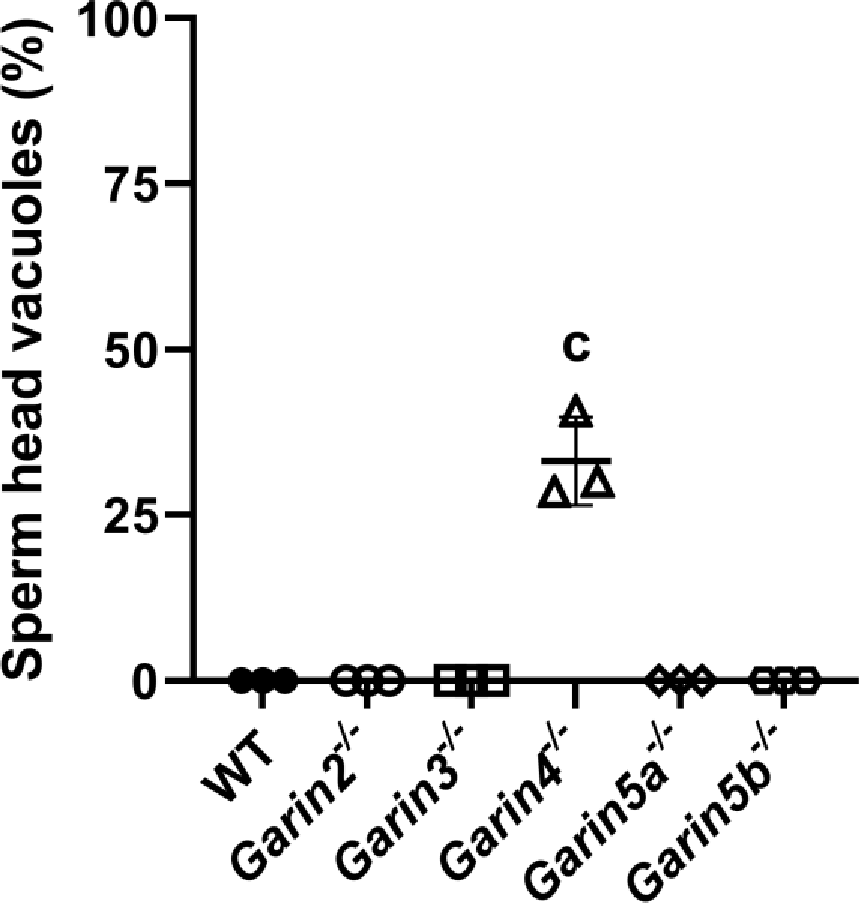

Supplement: S5 Fig — The percentage of vacuole-containing sperm heads were analyzed. Garin2-5b (Garin2, 3, 4, 5a, and 5b) KO males were compared with WT males (One-way ANOVA, c, P < 0.001). (TIF) [file pgen.1011337.s005.tif]

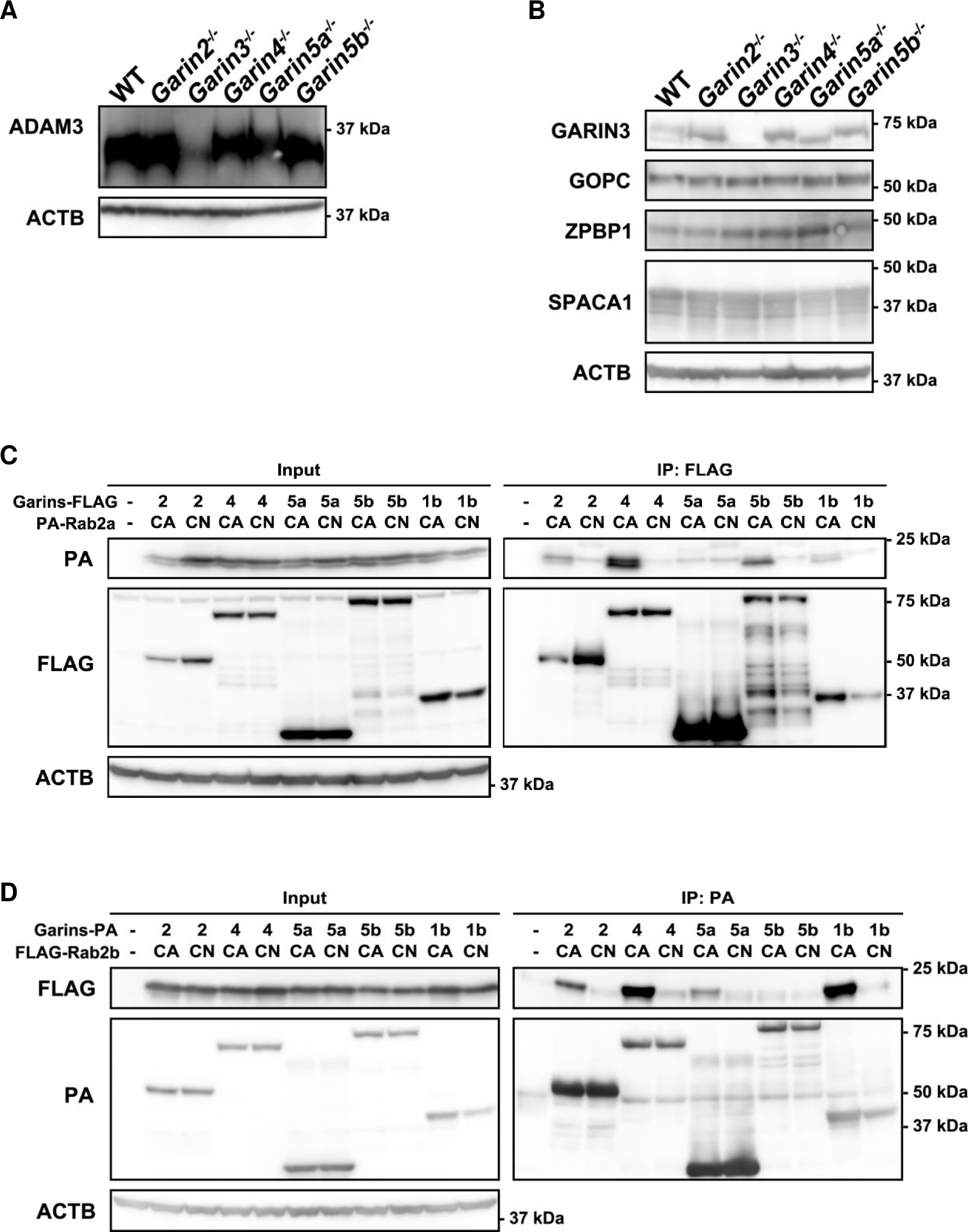

Supplement: S6 Fig — (A) Expression of ADAM3 in mature spermatozoa was analyzed using cauda epididymal sperm lysates from WT and Garin2-5b-/- males. (B) Western blotting analysis was performed using testis lysates from WT and Garin2-5b-/- males. Globozoospermia-related proteins, GOPC, ZPBP1, and SPACA1 were blotted with their corresponding antibodies. Anti-ACTB was used to detect ACTB as an endogenous control. (C and D) Co-IP of GARIN1B, GARIN2, GARIN4, GARIN5A, and GARIN5B with constitutively active (CA)/constitutively negative (CN) forms of RAB2A (C) or RAB2B (D). (TIF) [file pgen.1011337.s006.tif]
